# Evaluation of the Therapeutic Effects of Harmine on Anaplastic Thyroid Cancer Cells

**DOI:** 10.3390/ijms25021121

**Published:** 2024-01-17

**Authors:** Enke Baldini, Silvia Cardarelli, Antonio Francesco Campese, Eleonora Lori, Poupak Fallahi, Camilla Virili, Flavio Forte, Daniele Pironi, Filippo Maria Di Matteo, Piergaspare Palumbo, Maria Ludovica Costanzo, Vito D’Andrea, Marco Centanni, Salvatore Sorrenti, Alessandro Antonelli, Salvatore Ulisse

**Affiliations:** 1 Department of Surgery, “Sapienza” University of Rome, 00161 Rome, Italy; enke.baldini@uniroma1.it (E.B.); silvia.cardarelli@uniroma1.it (S.C.); eleonora.lori@uniroma1.it (E.L.); daniele.pironi@uniroma1.it (D.P.); filippomaria.dimatteo@uniroma1.it (F.M.D.M.); piergaspare.palumbo@uniroma1.it (P.P.); costanzomarialudovica@gmail.com (M.L.C.); vito.dandrea@uniroma1.it (V.D.); salvatore.sorrenti@uniroma1.it (S.S.); 2 Department of Molecular Medicine, “Sapienza” University of Rome, 00161 Rome, Italy; antonello.campese@uniroma1.it; 3 Department of Translational Research and New Technologies in Medicine and Surgery, University of Pisa, 56126 Pisa, Italy; poupak.fallahi@unipi.it; 4 Department of Medico-Surgical Sciences and Biotechnologies, “Sapienza” University of Rome, 04100 Latina, Italy; camilla.virili@uniroma1.it (C.V.); marco.centanni@uniroma1.it (M.C.); 5 Department of Urology, M.G. Vannini Hospital, 00177 Rome, Italy; flavioforte@hotmail.com; 6 Department of Surgical, Medical and Molecular Pathology and of Critical Area, University of Pisa, 56126 Pisa, Italy; alessandro.antonelli@unipi.it

**Keywords:** anaplastic thyroid cancer, epithelial mesenchymal transition, Twist1, harmine

## Abstract

Anaplastic thyroid carcinoma (ATC) is an extremely difficult disease to tackle, with an overall patient survival of only a few months. The currently used therapeutic drugs, such as kinase inhibitors or immune checkpoint inhibitors, can prolong patient survival but fail to eradicate the tumor. In addition, the onset of drug resistance and adverse side-effects over time drastically reduce the chances of treatment. We recently showed that Twist1, a transcription factor involved in the epithelial mesenchymal transition (EMT), was strongly upregulated in ATC, and we wondered whether it might represent a therapeutic target in ATC patients. To investigate this hypothesis, the effects of harmine, a β-carboline alkaloid shown to induce degradation of the Twist1 protein and to possess antitumoral activity in different cancer types, were evaluated on two ATC-derived cell lines, BHT-101 and CAL-62. The results obtained demonstrated that, in both cell lines, harmine reduced the level of Twist1 protein and reverted the EMT, as suggested by the augmentation of E-cadherin and decrease in fibronectin expression. The drug also inhibited cell proliferation and migration in a dose-dependent manner and significantly reduced the anchorage-independent growth of both ATC cell lines. Harmine was also capable of inducing apoptosis in BHT-101 cells, but not in CAL-62 ones. Finally, the activation of PI3K/Akt signaling, but not that of the MAPK, was drastically reduced in treated cells. Overall, these in vitro data suggest that harmine could represent a new therapeutic option for ATC treatment.

## 1. Introduction

Anaplastic thyroid carcinoma (ATC) is an infrequent but highly aggressive and commonly fatal thyroid cancer characterized by a rapid local progression and early establishment of distant metastases to lung, bone, liver, or brain [1,2]. ATC is usually observed in elderly patients, mostly aged over 60 years, whose median overall survival (OS) is a few months from the diagnosis [1,2,3,4]. It may originate as a primary tumor, i.e., ex novo in individuals without a history of thyroid neoplasms, or as a secondary tumor, arising from preexisting differentiated (DTC) or poorly differentiated (PDTC) thyroid carcinomas [1,4,5]. A recent study analyzing the Surveillance, Epidemiology, and End Result (SEER) database reported that ATC represents 1% of all thyroid cancers and that secondary ATC accounts for only 3.7% of all ATC [5]. Conversely, studies of single institutional experiences with small to moderate case series described an incidence of secondary ATC varying from 5 to 50% of all ATC [5,6,7,8]. At any rate, patients with primary and secondary ATC share very similar clinical features and overall survival [4,5,9].

Several molecular alterations have been involved in the etiopathogenesis of ATC [1,4]. The most frequent are those of the TERT promoter and TP53 gene encountered, respectively, in 75% and 63% of ATC patients [10,11]. Activating point mutations of the BRAF gene are also commonly met in ATC tissues, but their frequency varies in the different geographical areas from 14% to 45% [10,11,12]. Of note is that BRAF alterations are found more often in secondary ATC tumors, containing foci of DTCs, compared with primary ATCs [1,13,14]. Other molecular alterations encountered in ATC are gene mutations of RAS in ~22% of cases, PIK3CA in ~18% of cases, EIF1AX and PTEN in ~14% of cases, and NTRK and RET fusions in 2–3% of cases [10,11,15,16,17]. The aberrant expression of genes involved in Wnt signaling, DNA mismatch repair, the cell cycle, matrix remodeling enzymes, and different epigenetic processes have also been described in ATC tissues with variable frequencies [10,11,17,18,19,20,21,22,23,24]. However, a deeper comprehension of the molecular mechanisms responsible for ATC progression is of paramount importance for this deadly tumor, especially in this era, where a growing number of targeted therapies are being developed. This issue was highlighted in the American Thyroid Association (ATA) and European Society for Medical Oncology (ESMO) guidelines, which recommended performing molecular tests for ATC patients with unresectable diseases [3,25,26].

Type III epithelial–mesenchymal transition (EMT) is a hallmark of cancer that plays a pivotal role in malignant cells dissemination [27,28,29]. The transition from an epithelial to a mesenchymal phenotype grips a variety of cellular changes, not all of which necessarily occur. Instead, tumor cells infrequently undertake a complete EMT, acquiring some mesenchymal characteristics while maintaining epithelial features [27,28,29,30,31]. The skill of cancer cells to attain a mixed epithelial–mesenchymal phenotype, along with their ability to move along the epithelial–mesenchymal spectrum, is denoted as the epithelial–mesenchymal plasticity (EMP) [32]. The EMT degree affects the tumor metastatization mode, with cells characterized by partial EMT spreading in multicellular clusters, while those with a complete EMT migrate to distant sites as single cells [33]. Different players within the primary tumor microenvironment are assumed to affect cancer cells’ EMT, including cellular and humoral components of inflammation, hypoxia, extracellular matrix enzymes, and available growth factors [28]. All of these have been shown to modulate the expression of EMT transcription factors (EMT-TFs), namely Zeb1 and Zeb2, Snail1 and Snail2, and Twist1 [28]. These EMT-TFs repress the expression of epithelial markers (i.e., E-cadherin, claudin, occludin) while prompting that of mesenchymal genes (i.e., N-cadherin, vimentin, fibronectin) [28]. In addition, a number of experimental findings have suggested that the role of EMT-TFs goes beyond cancer cell metastatization, controlling processes such as cell fate specification, malignant transformation, cancer stem cell plasticity, resistance to therapy, and immune evasion [34]. Altogether, these observations indicate that EMT-TFs could represent new targets for the treatment of aggressive cancers, including ATC.

We recently showed that, among the five EMT-TFs above mentioned, Twist1 was the only one to be strongly upregulated in ATC tissues compared with normal thyroid or papillary thyroid cancer (PTC) tissues [35]. This evidence is corroborated in earlier studies showing higher Twist1 protein levels in ATC compared with normal thyroid and DTC and the ability of the Twist1/miR-584/TUSC2 pathway to induce resistance to apoptosis in thyroid cancer cells [36,37]. In addition, it has been demonstrated that the most upregulated genes induced by Twist1 in thyroid cancer cells are those controlling motility, proliferation, cell death, and survival [38]. Interestingly, the knockdown of Twist1 by RNA interference in ATC cells reduced cell migration and invasion and increased sensitivity to apoptosis [37]. Similar effects were obtained in the PTC-derived TPC-1 cell line following treatment with harmine, a beta-carboline alkaloid of plant origin having a broad spectrum of anti-inflammatory and antitumor activities capable of inhibiting Twist1 protein expression by promoting its degradation [36,39,40]. Altogether, these observations suggest that Twist1 could represent a valuable molecular therapeutic target in ATC patients.

Therefore, in the present study, we sought to evaluate the effects of harmine on growth, motility, survival, and the epithelial–mesenchymal transition state of two ATC-derived cell lines at the preclinical level.

## 2. Results

### 2.1. Harmine’s Effects on Twist1 Expression and EMT Markers in ATC-Derived Cell Lines

**Figure 1:**
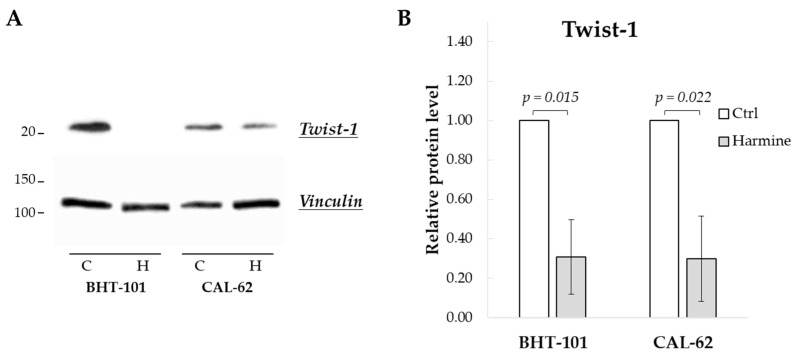
Harmine-reduced Twist1 protein level in ATC-derived cell lines. Cells were incubated for 48 h with or without 20 μM harmine, after which cell protein extracts were prepared. (**A**) Western blotting image of Twist1 and vinculin (loading control). (**B**) Densitometric analysis of the results. Bars represent the mean ± SEM (standard error of the mean) of three independent experiments. C, control; H, harmine.

We first evaluated the effect of harmine exposure on Twist1 protein levels of BHT-101 and CAL-62 cells. As shown in Figure 1, treatment with 20 μM harmine for 48 h considerably reduced Twist1 protein amount by an average of 70% in both ATC cell lines. This dose also inhibited cell growth by 50% or more in both cell lines as described below, and thus, it was used in most of the experiments.

**Figure 2:**
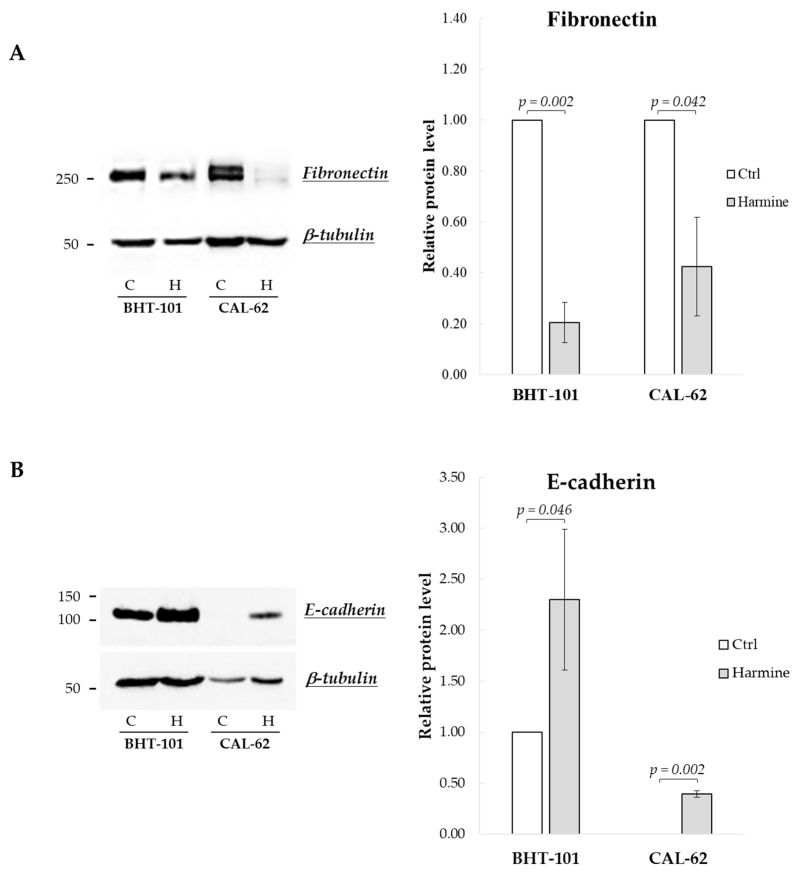
Effects of harmine on E-cadherin and fibronectin protein levels in ATC-derived cell lines. BHT-101 and CAL-62 were incubated, respectively, for 72 h and 96 h with or without 20 μM harmine. At the end of the incubation, protein extracts were prepared and analyzed. Western blotting and densitometric analyses for fibronectin (**A**) and E-cadherin (**B**). Bars represent the mean ± SEM of three independent experiments. C, control; H, harmine.

We then examined harmine’s effects on the expression of EMT markers, i.e., E-cadherin and fibronectin. As reported in Figure 2, both BHT-101 and CAL-62 cells showed increased levels of E-cadherin and a reduction in fibronectin following treatment with 20 μM harmine for 72 h and 96 h, respectively.

### 2.2. Harmine’s Effects on ATC Cell Migration

**Figure 3:**
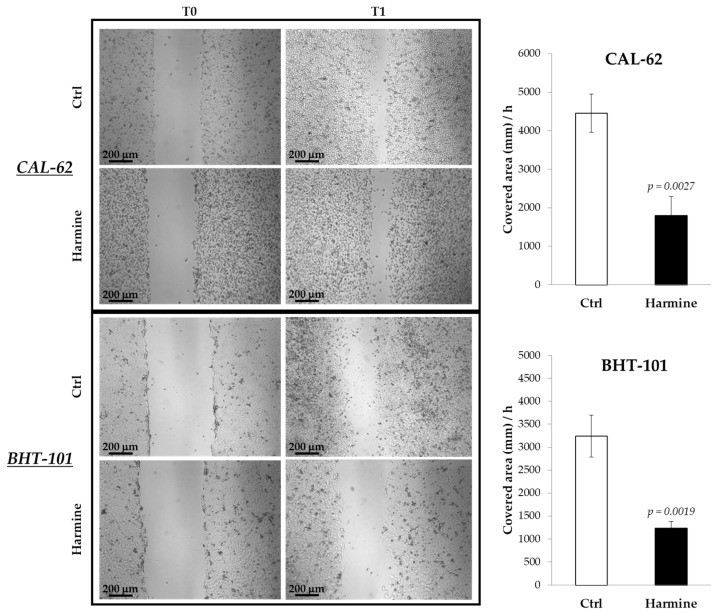
Effects of harmine on ATC cell migration in adherent cultures. Cells were seeded on dishes and preincubated overnight with or without 20 μM harmine. Scratches were created on 100% confluent cultures, and fresh medium ± harmine was added. Dishes were photographed immediately after the scratch and at different time intervals. The closure time of the scratch was calculated with ImageJ software. Bars represent the mean ± standard deviation (SD) of three independent experiments.

The effects of harmine on the motility of ATC cells were investigated in both 2D and 3D cultures by means of a scratch assay and spheroid spreading on an adherent surface. The results obtained from the scratch assay, reported in Figure 3, demonstrated the ability of harmine to significantly reduce the migration of CAL-62 and BHT-101 in monolayer cultures.

**Figure 4:**
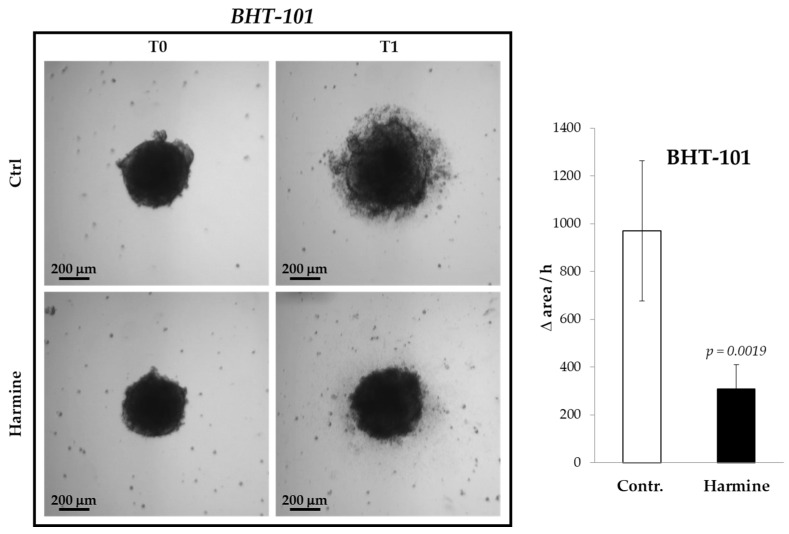
Effects of harmine on BHT-101 spheroid spreading. Preformed spheroids were seeded onto adherent dishes and incubated with or without 20 μM harmine. Dishes were photographed immediately and at different time intervals for 24 h. The area occupied by disseminating cells was measured by ImageJ software. Bars represent the mean ± SD of three independent experiments.

The inhibitory effect of harmine on the motility of ATC cells was confirmed in 3D cultures, that is, in spheroids formed by BHT-101 cells onto a poly-HEMA substrate. It was not possible to perform such an experiment with CAL-62 because these cells were not capable of generating spheroids but merely aggregated into poorly compacted clusters. As evident from Figure 4, treatment with harmine significantly increased the spreading rate of BHT-101 spheroids placed on an adherent surface.

### 2.3. Harmine’s Effects on ATC Cells Proliferation and Anchorage-Independent Growth

**Figure 5:**
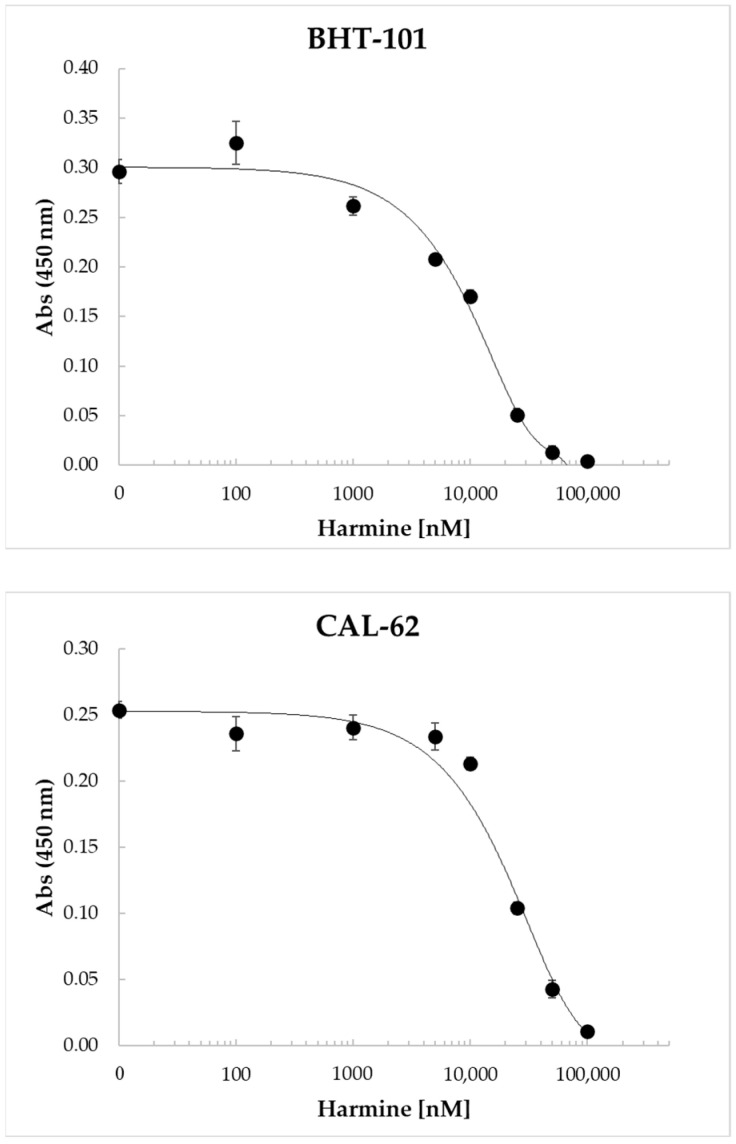
Dose-dependent inhibition of BHT-101 and CAL-62 proliferation by harmine. ATC cells were seeded in 96-well plates in quadruplicate and treated with increasing concentrations of harmine (0.1–100 μM) for 72 h. At the end of the incubation time, the WST-1 reagent was added to each well, and the absorbance was read after 4 h with a microplate ELISA reader. Points are means and error bars are SD of quadruplicate wells.

Harmine and its benzo[d]imidazo[2,1-b]thiazole derivatives were found to inhibit the proliferation of a number of human cell lines derived from different tumor types, including colon, liver, breast, and lung cancers [40,41,42]. Therefore, we investigated the dose-dependent effects of harmine on ATC cell proliferation. The results of these experiments, reported in Figure 5, clearly demonstrated the ability of the drug to inhibit cell proliferation in a dose-dependent manner, with an IC_50_ of 11.7 ± 308 μM for BHT-101 and 22.0 ± 1.6 μM for CAL-62.

**Figure 6:**
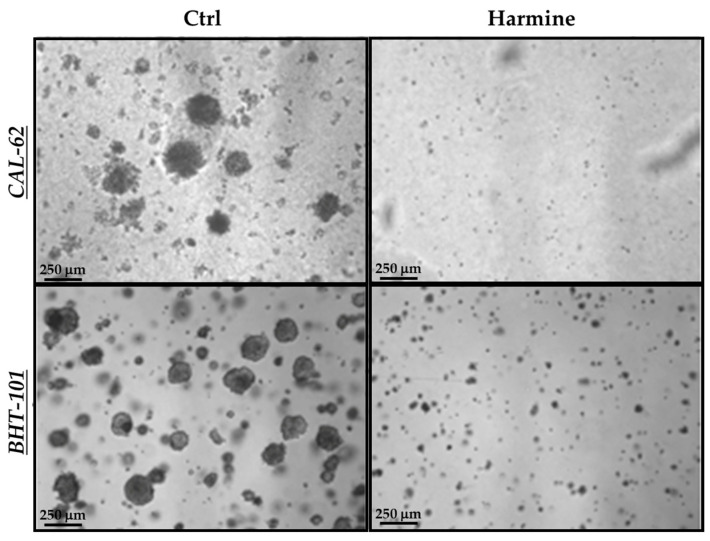
Effects of harmine on the anchorage-independent growth of ATC cells. Cells were plated in soft agar medium containing 50 μM harmine or a vehicle alone and incubated for 10–15 days. Photos of each treatment and control were acquired, and colonies with a ≥50 μm diameter were counted.

In view of the above results, we next evaluated harmine’s effects on the anchorage-independent growth of ATC cells in a semisolid milieu. As reported in Figure 6, exposure to 50 μM harmine totally impaired the ability of both cell lines to form colonies in soft agar after 10–15 days of incubation.

### 2.4. Harmine’s Effects on ATC Cells Apoptosis

**Figure 7:**
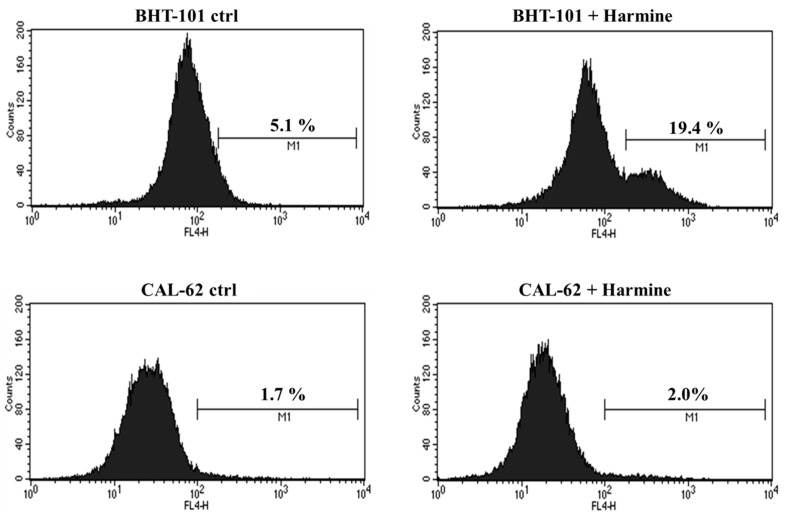
Effects of Harmine on ATC cell apoptosis. Cells were cultured with or without 20 μM harmine for 48 h; then, they were stained for annexin V and analyzed by cytofluorimetry. M1: Annexin V positive cells.

Moreover, we evaluated whether harmine was capable of inducing apoptosis in the two ATC cell lines. To this end, cells were cultured in the absence or presence of 20 μM harmine for 48 h and was then stained for annexin V and analyzed by cytofluorimetry. The results, shown in Figure 7, demonstrated that harmine triggered apoptosis in BHT-101 cells but not in CAL-62 cells. No signs of cell death were observed in CAL-62, even at the 50 μM concentration of harmine, which resulted in a growth inhibition of almost 90%.

**Figure 8:**
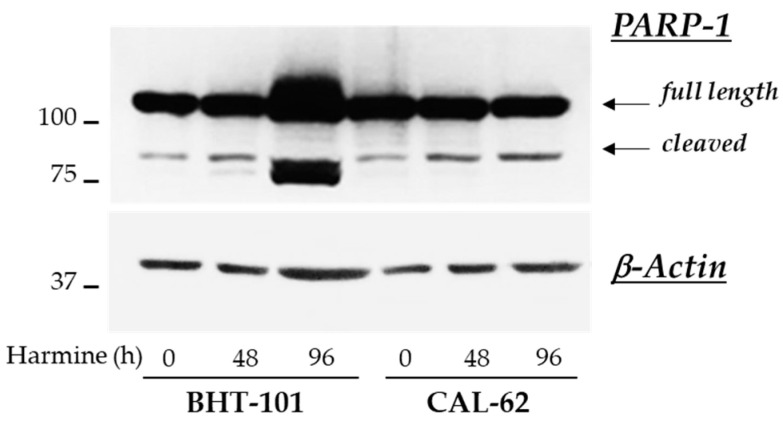
Effects of harmine on PARP-1 cleavage in ATC-derived cell lines. Cells were cultured for 48 h or 96 h with or without 20 μM harmine. At the end of the incubation time, cells were harvested and protein extracts were prepared. PARP-1 and β-actin protein levels were analyzed by Western blotting.

These observations were corroborated by the analysis of further apoptotic markers in control and treated cells. Unexpectedly, no fragmentation of caspases 3, 8, and 9 was detected by Western blot experiments in either ATC cell lines; however, 20 μM harmine strongly induced the cleavage of PARP-1 in BHT-101 cells (see Figure 8).

### 2.5. Harmine Effects on the MAPK and PI3K/Akt Signaling Pathways in ATC Cells

**Figure 9:**
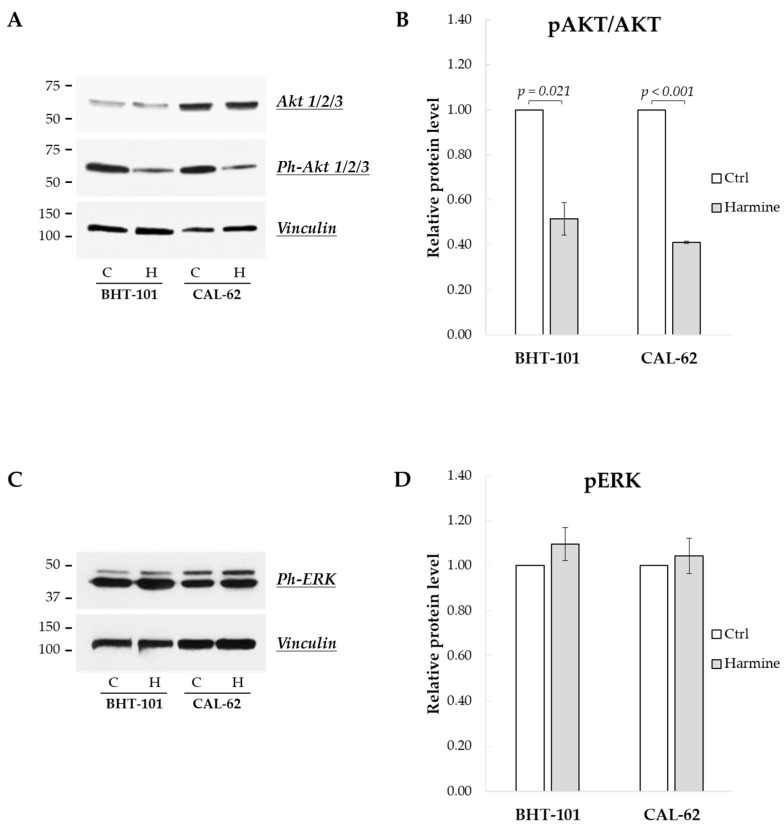
Effects of harmine on the phosphorylation status of Akt and ERK kinases in ATC-derived cell lines. Cells were incubated for 24 h or 48 h with or without 20 μM harmine; then, they were harvested, and the cell protein extracts were prepared. Ph-Akts, Akts, Ph-ERKs, and vinculin protein levels were analyzed by Western blotting (**A**,**C**). The densitometric analyses, reported in (**B**,**D**), were performed on a minimum of three independent experiments. Bars represent the mean ± SEM. C, control; H, harmine.

Finally, we sought to investigate the effects of harmine on the MAPK and PI3K/Akt signaling pathways, frequently deregulated in ATC [24]. As reported in Figure 9A,B, the phosphorylation status of Akt1/2/3 kinases was drastically reduced following 24 h of treatment with harmine, while the phosphorylation of ERK1/2 kinases was unaffected, even after 48 h (see Figure 9C,D).

## 3. Discussion

Anaplastic thyroid cancer (ATC) represents a rare highly aggressive thyroid cancer with very limited therapeutic options and a disease-specific mortality of about 99% [1,3,25,43,44,45,46,47,48,49,50,51,52]. The strategies currently implemented include surgery, usually limited to patients with localized disease, followed by adjuvant radio-, chemo-, immuno-, and/or targeted therapies [3,52]. In particular, over the last few years, the advancement of knowledge on ATC genomic alterations has led to the clinical trials of a number of molecular targeted drugs [51]. Among these, small molecule inhibitors targeting BRAF (dabrafenib) and MEK (trametinib) exhibited outstanding responses in ATC patients carrying the BRAF^V600E^ mutation [3,52,53,54,55,56]. However, effective therapies for patients harboring the wild type BRAF are still lacking.

Harmine, a β-carboline alkaloid drug isolated from the seeds of the medicinal plant Peganum harmala, has been shown to possess antitumor activities both in vitro and in vivo in different cancer types, as well as in a PTC-derived cell line (TPC-1) [39,41]. In particular, harmine was found to inhibit the activity of some enzymes, e.g., monoamine oxidases, topoisomerases, and the dual-specificity tyrosine phosphorylation-regulated kinase 1A (DYRK1A); moreover, to date, it is the only known pharmacological inductor of Twist1 protein degradation.

Based on previous findings that Twist1 is highly expressed in ATC tissues, in the present study, we investigated the effects of harmine on ATC cell growth, survival, and tumorigenicity in a preclinical setting [35,36,37,38]. Our results evidenced that Twist1 inhibition by harmine promoted the reversal of the EMT transition in ATC cells, as indicated by the increase in E-cadherin expression and concomitant reduction in fibronectin expression, as well as through the delayed cell migration of both ATC cell lines. These data are in line with those of previous studies describing the ability of harmine to inhibit the migration and invasion of a number of cell lines derived from melanoma, glioblastoma, neuroblastoma, leukemia, bladder, ovarian, thyroid, and pancreatic cancers [39,41,57,58,59,60,61,62,63,64,65]. Furthermore, harmine was capable of inhibiting in a dose-dependent manner the proliferation of BHT-101 and CAL-62 cells with an IC_50_ comparable with those previously reported for other tumor-derived cell lines [39,41,57,58,59,60,61,62,63,64,65].

The PI3K/Akt and MAPK signaling pathways are strongly activated in thyroid cancer progression, prompting cell proliferation and survival [1,2,3,4]. Previous works demonstrated that harmine significantly reduced the level of Akt phosphorylation in gastric, colon, and breast cancer-derived cell lines, as well as ERK phosphorylation in colon and breast cancer cell lines [41]. Similarly, we observed that harmine caused a strong decrease in Akt phosphorylation in both ATC cells, thus attenuating the PI3K/Akt signaling. In our experimental setting, we failed to observe any modifications of ERK phosphorylation status. Nonetheless, it can be assumed that interference with the Akt signal, together with the harmine aptitude to bind to DNA and inhibit the activity of the topoisomerase I, most likely contribute to the antiproliferative effect observed in both cell lines [41]. In this context, it is of interest to note that several derivatives of harmine, especially those with substitution in positions 1 and 9 of the molecule, displayed higher DNA binding affinity and antiproliferative effects with lower IC_50_ values in several tumor-derived cell lines [42,66,67,68,69,70,71]. In our experimental model, treatment with harmine was also able to drastically inhibit the anchorage-independent growth of both ATC cell lines, corroborating previous results obtained in a PTC cell line [39]. The activation of caspases and apoptosis induction was another feature commonly encountered in tumor-derived cell lines following harmine exposure [41,57,58,59,60,61,62,63,64,65]. We did not observe fragmentation of any caspases, although annexin V labeling and PARP-1 cleavage were evident in BHT-101 cells. These apparently conflicting results could be explained considering that PARP-1, in addition to caspases, can be cut by various proteins, including lysosomal cathepsins [72]. Harmine was found to induce lysosomal membrane permeabilization and the consequent release of enzymes into the cytosol, activating the so-called lysosomal apoptotic pathway [73]. It can be hypothesized that in BHT-101, apoptosis occurs through a caspase-independent pathway(s), possibly involving lysosomal proteases translocated to the cytosol, which are responsible for PARP-1 cleavage.

We are aware that this study suffers from some evident limitations. First, harmine is a compound that is active on various molecular substrates, each of which regulates different biochemical pathways, and broader and more in-depth analyses are required to elucidate the molecular mechanisms elicited by harmine in ATC and identify its most relevant therapeutic targets. Such knowledge would enable the design and selection of more powerful and specific pharmacological derivatives for anti-ATC therapy. Unlike BHT-101 cells, CAL-62 cells did not undergo apoptosis following treatment; thus, it would be especially interesting to determine which factors are responsible for this resistance. It can be hypothesized, for instance, that the presence of a gain-of-function mutation of the KRAS gene in CAL-62 and/or of the BRAF^V600E^ mutation in BHT-101 play a role. Moreover, in vivo experiments are needed to verify whether and to what extent harmine is able to counteract ATC growth and spread in living organisms.

Notwithstanding the requirement of further studies, the data reported here suggest the potential usefulness of harmine, and eventually its derivatives, as therapeutic agents for the treatment of ATC.

In conclusion, the present work documented the ability of harmine to exert multiple anti-cancer actions on ATC cells, being able to revert the EMT by reducing the expression of Twist1; and to impair ATC cell proliferation, motility, anchorage-independent growth and, in one ATC cell line, survival.

## 4. Materials and Methods

### 4.1. Cell Cultures

The ATC-derived cell lines BHT-101 and CAL-62 were purchased from DSMZ (Braunschweig, Germany), and cultured in DMEM containing 10% (CAL-62) or 20% (BHT-101) fetal bovine serum (FBS) and 2 mM L-glutamine at 37 °C in a 5% CO_2_ humidified atmosphere. Harmine (7-methoxy-1-methyl-9H-pyrido[3,4-b]indole) was acquired in powder form (Merck Life Science S.r.l., Milan, Italy), and dissolved in DMSO before use. In all the experiments, control cells were treated with the drug vehicle alone at the same volume of the highest dose of harmine employed. Culture medium containing or not containing the drug was changed every other day up to the end of the incubation time.

### 4.2. Western Blot

Cells treated with or without harmine were lysed in RIPA buffer with fresh added protease and phosphatase inhibitors. A total of 30 μg of proteins were run in SDS-PAGE under reducing conditions and blotted onto nitrocellulose membranes. These were saturated for 2 h in TBS-Tween with 5% nonfat dry milk, then incubated overnight at +4 °C with the primary antibodies anti-Twist1 1:1000 (ab50581, Abcam Inc., Cambridge, MA, USA), anti-PARP-1 1:2000 (sc-7150, Santa Cruz Biotechnology, Inc., Heidelberg, Germany), anti-E-cadherin 1:1000 (3195, Cell signaling Technology, Danvers, MA, USA), anti-fibronectin 1:2000 (ab2413, Abcam, Cambridge, UK), anti-phospho(Thr308) AKT1/2/3 1:500 (sc-16646, Santa Cruz Biotechnology, Inc., Heidelberg, Germany), anti-AKT1/2/3 1:500 (sc-8312, Santa Cruz Biotechnology, Inc., Heidelberg, Germany), anti-phospho (Thr 202/204) ERK1/2 1:1000 (sc-16982, Santa Cruz Biotechnology, Inc., Heidelberg, Germany), anti-β-actin 1:10000 (A2066, Sigma-Aldrich/Merk Life Science S.r.l., Milan, Italy), and anti-vinculin 1:10000 (ab129002, Abcam Inc., Cambridge, MA, USA). After washing, membranes were incubated with appropriate HRP-conjugated secondary antibodies diluted 1:10000 (Thermo Fisher Scientific, Rockford, IL, USA) and were developed using the LiteAblot EXTEND chemiluminescent substrates (Euroclone, Milan, Italy). Immunopositive bands were detected with the iBright1500 instrument (Thermo Fisher Scientific, Waltham, MA, USA) and quantified by scanning densitometry using the iBright analysis software (version 5.2).

### 4.3. Migration Assay

ATC cells were seeded onto 60 mm dishes in medium ± harmine (20 μM) so as to obtain 100% confluence the day after. Three scratches per dish were created in the cell monolayer with a p200 pipette tip. After washing to remove debris, fresh medium ± harmine was added, and image fields for capturing were marked by reference signs. The dishes were photographed immediately with the Moticam 2500 digital camera connected to the microscope Motic-BA410 (Motic, Barcelona, Spain), then placed in an incubator and photographed again at different time intervals. Finally, the closure time of the scratches was calculated for each culture using ImageJ software (version 1.48).

### 4.4. Spheroid Formation and Spreading

ATC cells were seeded in round bottom 96-well plates previously coated with PolyHEMA (Poly(2-hydroxyethyl methacrylate)) hydrogel (Merck Life Science S.r.l., Milan, Italy). Plates were centrifuged for 6 min at 232 g to bring the cells together and then incubated in standard conditions. Culture media were changed every 48 h, and spheroid formation occurred after 3–4 days of incubation. On the fifth day, fresh medium ± 20 μM harmine was added to half of the wells. After 24 h incubation, spheroids were individually aspirated and transferred into 24-well adherent plates with the same culture medium. Spheroids were photographed immediately (T0) and after about 24 h (T1) with a microscope digital camera. The surface occupied by cells was measured using ImageJ software, and the spreading rate was calculated as (area T1 − area T0)/time.

### 4.5. Proliferation Assay

ATC cells were seeded in 96-well plates (2000 cells/well) in quadruplicate. The day after, cells were treated with increasing doses of harmine (from 0.1 to 100 μM) for 72 h, changing media ± harmine after 48 h. Finally, the tetrazolium salt WST-1 was added to each well, and the absorbance was read 4 h later using a microplate ELISA reader (Tecan Group Ltd., Männedorf, Switzerland). IC_50_ values were calculated for each cell line by using the MyCurveFit online tool (https://mycurvefit.com/, accessed on 18 November 2023).

### 4.6. Colony Formation in Soft Agar

Petri dishes containing soft agar cell cultures supplemented with 20 μM harmine or a vehicle alone were prepared as previously described [74]. After 2 weeks of incubation, the dishes were photographed. Nine fields were acquired for each dish, and pictures were analyzed by means of the ImageJ program, scoring colonies with a diameter of ≥50 μm.

### 4.7. Cytofluorimetric Analysis of Apoptosis

ATC cells were cultured with or without harmine for 48 h; then, they were harvested by scraping in PBS, washed and centrifuged twice, and counted. Apoptotic cells were marked by staining with allophycocyanin (APC) annexin V following the manufacturer’s instructions (BD Biosciences, Franklin Lakes, NJ, USA); finally, the cells were analyzed by FACS using the FACScalibur flow cytometer and CeCellQuestPro software (version 6) (BD Biosciences, San Jose, CA, USA) within 1 h. Dead cells were excluded from the analysis by gating the viable cell population on the basis of physical parameters (FSC/SSC).

### 4.8. Statistical Analysis

For cell migration, spheroid spreading, and colony formation assays, the *t*-test was used to assess differences between control and treated cells. The Kruskal–Wallis and Bonferroni post hoc tests were applied to the analysis of dose–response data. All statistics were performed with SPSS software 25 (Armonk, NY, USA), and the results were considered significantly different when the pertaining *p*-values were <0.05.

## Data Availability

Data and information related to this study are available upon request.
